# Racial and Ethnic Differences in Mental Health Service Use Among Adolescents

**DOI:** 10.1001/jamanetworkopen.2025.16612

**Published:** 2025-06-18

**Authors:** Yanlei Ma, Cristian Ramos, Hefei Wen, Janet R. Cummings

**Affiliations:** 1 Harvard T.H. Chan School of Public Health, Boston, Massachusetts; 2 Rollins School of Public Health, Emory University, Atlanta, Georgia; 3 Harvard Medical School, Boston, Massachusetts; 4 Harvard Pilgrim Health Care Institute, Boston, Massachusetts

## Abstract

**Importance:**

The delivery of mental health services to US adolescents has changed significantly in recent years, especially with the increased use of telemental health. More data are needed to understand the implications for racial and ethnic differences in adolescent mental health care use.

**Objective:**

To examine racial and ethnic differences in mental health service use among US adolescents in the most recent national survey data.

**Design, Setting, and Participants:**

This cross-sectional study used 2022 to 2023 US national survey data to estimate racial and ethnic differences in mental health service use through weighted logistic regressions, controlling for sociodemographic characteristics and health status. Adolescents participating in the National Survey on Drug Use and Health in 2022 to 2023 were included.

**Exposure:**

Racial and ethnic group.

**Main Outcomes and Measures:**

Dichotomous indicators of mental health treatment type included receipt of any mental health visit (medical setting), prescription medication, support group, or peer support specialist or recovery coach service. Treatment settings were assessed with indicators of mental health services received in outpatient, telehealth, school, inpatient, and emergency department settings.

**Results:**

The overall sample included a total of 23 541 adolescents aged 12 to 17 years (8351 aged 14-15 years [weighted percentage = 35.1%]; 12 167 male [weighted percentage = 51.1%]; 6057 Hispanic [weighted percentage = 26.0%], 1202 non-Hispanic Asian, Hawaiian, or Other Pacific Islander [weighted percentage = 6.2%], 3239 non-Hispanic Black [weighted percentage = 13.8%], 10 756 non-Hispanic White [weighted percentage = 49.7%], and 2287 other non-Hispanic race or ethnicity [weighted percentage = 4.3%]), of whom 5994 individuals had experienced a major depressive episode. The model-adjusted percentage of adolescents receiving any mental health visit was 31.7% (95% CI, 30.4%-33.1%) among non-Hispanic White adolescents and was significantly lower among members of racial and ethnic minority groups, ranging from 21.9% (95% CI, 19.5%-24.3%) among non-Hispanic Black adolescents to 25.6% (95% CI, 23.6%-27.6%) among Hispanic adolescents (all *P* < .001). Significant differences were also seen in prescription medication use and receipt of care in outpatient, school, and telemental health settings. For example, 17.0% (95% CI, 16.0%-18.0%) of non-Hispanic White adolescents received a telemental health visit after adjustment, but this percentage ranged from 8.1% (95% CI, 5.4%-10.8%) among non-Hispanic Asian, Hawaiian, or Other Pacific Islander adolescents to 12.0% (95% CI, 10.4%-13.6%) among Hispanic adolescents (all *P* < .001). Few to no racial or ethnic differences were observed in receipt of mental health services from support groups, peer support specialists or recovery coaches, inpatient or residential settings, or emergency departments. Similar patterns were observed among adolescents with past major depressive episodes.

**Conclusions and Relevance:**

In this study, substantial racial and ethnic differences were observed in US adolescent mental health service use after the COVID-19 pandemic, particularly in psychotropic medication use and clinical outpatient, school, and telemental health settings. These findings highlight the need to improve mental health access for adolescent members of racial and ethnic minority groups.

## Key Points

**Question:**

How does mental health service use differ among US adolescents across racial and ethnic groups after COVID-19?

**Findings:**

In this cross-sectional study of 23 541 adolescents, members of racial and ethnic minority groups were significantly less likely to access mental health visits or receive psychotropic medications or services in outpatient, telemental health, or school settings compared with White adolescents. Few to no racial or ethnic differences were observed in use of support groups, peer support, or services in emergency department or inpatient settings.

**Meaning:**

In this study, substantial racial and ethnic differences were observed in US adolescent mental health service use after the COVID-19 pandemic, highlighting the need to improve mental health access for adolescent members of minority populations.

## Teaser

This cross-sectional study investigates differences in mental health service use by race and ethnicity among adolescents after the COVID-19 pandemic.

## Introduction

The prevalence of mental health disorders among adolescents has been increasing in recent years across all racial and ethnic groups.^1,2,3,4^ Due to the worsening state of child and adolescent mental health, the American Academy of Pediatrics, American Academy of Child and Adolescent Psychiatry, and Children’s Hospital Association declared a national emergency to address this crisis.^5^ The declaration called for systemic reforms and increased investment in youth mental health, including increased access to services and funding for mental health programs and research.^5^

Racial and ethnic disparities in mental health service use among adolescents have been documented long before the COVID-19 pandemic.^6,7^ Prior studies examining trends in mental health service use by race and ethnicity reported that these disparities persisted over time^8^ or, in some cases, became exacerbated.^9^ For example, between 2010 and 2017, the percentage of White children and adolescents using any mental health care increased from 12.9% to 15.0%, while it decreased for Black children from 9.1% to 8.0%.^9^

In recent years, there have been significant changes in the delivery of mental health services for adolescents, catalyzed by the COVID-19 pandemic.^10,11^ One change to mental health service delivery in the past decade has been the increased government investment in school mental health services.^12,13^ Importantly, some evidence suggests that racial and ethnic differences may be less pronounced in school settings than in traditional, clinic-based settings.^6,14^ Another major change to the delivery system has been the dramatic increase of telemental health services among youths shortly after the onset of the pandemic.^15,16,17^ However, to date, there are limited data on racial and ethnic differences in adolescent use of telemental health services. A 2025 study^18^ found that among all Medicaid-enrolled children and adolescents (ages 3-17 years) with a mental health encounter, Asian, Black, and Hispanic children and adolescents were less likely than non-Hispanic White youths to receive services via telehealth. However, more data on racial and ethnic differences in telemental health use are needed beyond children and adolescents participating in the Medicaid program.

Given these significant changes to the delivery of mental health services to US adolescents, it is essential to understand the implications for racial and ethnic differences in mental health care use in this population. To address this gap in the literature, this study used the latest data from the 2022 and 2023 National Survey on Drug Use and Health (NSDUH) to examine racial and ethnic differences in adolescent mental health service use across treatment types and settings for the general adolescent population and adolescents with a past major depressive episode.

## Methods

The Emory Institutional Review Board (IRB) determined that this cross-sectional study was not human participants research because it involved the analysis of existing, publicly available data without any individual identifiers; thus, no IRB review was required and the requirements for informed consent under the Common Rule do not apply. The study is reported following the Strengthening the Reporting of Observational Studies in Epidemiology (STROBE) reporting guideline.

### Data

Data from 2022 and 2023 were pooled from the NSDUH, a nationally representative survey on behavioral health among the US civilian, noninstitutionalized population aged 12 years or older (see eAppendix 1 in Supplement 1 for survey questions). The analytic sample included 23 541 adolescents aged 12 to 17 years, of whom 5994 adolescents had experienced a major depressive episode in their lifetime based on *Diagnostic and Statistical Manual of Mental Disorders* (Fifth Edition) (*DSM-5*) criteria as assessed in the survey (see eAppendix 2 in Supplement 1 for detailed questions).^19^ We excluded 798 adolescents with missing data on major depressive episode status from analyses involving the subsample of adolescents with a major depressive episode.

### Measures

#### Mental Health Treatment Type

We created 4 dichotomous measurements of the type of mental health services that adolescents received in the past year: (1) any mental health visit in a medical setting (outpatient; telehealth, including phone or video; inpatient; and emergency room or department), (2) any mental health prescription medication, (3) any mental health support group, and (4) any help from a peer support specialist or recovery coach. These measures are not mutually exclusive given that an adolescent may have received multiple types of mental health treatment.

#### Mental Health Treatment Setting

We created 5 dichotomous measurements to assess whether adolescents received any past-year mental health treatment in the following settings: (1) outpatient (including outpatient mental health treatment center; outpatient drug or alcohol treatment or rehabilitation center; office of a therapist, psychologist, psychiatrist, or other mental health professional; general medical clinic or doctor’s office; outpatient hospital; and other), (2) telehealth (including phone or video), (3) school, (4) inpatient (including hospital, residential mental health treatment center, residential drug or alcohol rehabilitation or treatment center, and other), and (5) emergency room or department. These measures are not mutually exclusive given that an adolescent may have received treatment in multiple settings.

#### Race and Ethnicity

We categorized self-reported race and ethnicity into 5 mutually exclusive categories: (1) Hispanic; (2) non-Hispanic Asian, Hawaiian, or Other Pacific Islander; (3) non-Hispanic Black; (4) non-Hispanic White; and (5) other non-Hispanic race or ethnicity. The other non-Hispanic race or ethnicity comprised non-Hispanic adolescents who reported more than 1 racial background, as well as Native American or Alaska Native adolescents. We combined the non-Hispanic Asian category with the non-Hispanic Native Hawaiian or Other Pacific Islander category due to a small sample size for each group. Similarly, we combined the non-Hispanic Native American or Alaska Native category with the non-Hispanic more than 1 race category due to a small sample size for each group.

#### Sociodemographic and Health Status Measures

Our analysis controlled for additional predisposing, enabling, and need-related characteristics that may be associated with race and ethnicity and mental health service use (eTable 1 in Supplement 1).^20^ Predisposing characteristics included a categorical measure of age, an indicator for female vs male sex, and a categorical measure of household structure. Enabling characteristics included a categorical measure of family income, a categorical measure of current health insurance status (private insurance, Medicaid, other insurance, or uninsured), a categorical measure of English proficiency (speaking very well, speaking not very well, and unknown or missing), and a categorical measure of the metropolitan status of the county in which the adolescent resides. Need-related characteristics included indicators for a past major depressive episode, fair or poor self-rated overall health status, the presence of any self-reported chronic condition (including asthma, cancer, chronic bronchitis or chronic obstructive pulmonary disease, cirrhosis, diabetes, heart condition, hepatitis B or hepatitis C, high blood pressure, HIV/AIDS, and kidney disease), and the presence of a drug or alcohol use disorder in the past year.

### Statistical Analysis

For the overall sample of adolescents and the subsample with a past major depressive episode, unweighted sample sizes and weighted percentages were provided for all covariates. Bivariate comparisons were made between each minority racial and ethnic group and non-Hispanic White adolescents for all covariates using weighted Wald tests. We also compared percentages of adolescents who used each type of mental health service or any service in a given setting by race and ethnicity using weighted Wald tests.

Multivariate analyses were conducted with pooled weighted logistic regression models for the overall sample of adolescents and the subsample with a past major depressive episode. The dependent variables in our regression analyses were mental health treatment type or mental health treatment setting. The key independent variable was our categorical measure of race and ethnicity. We also controlled for confounding predisposing, enabling, and need-related measures, as well as survey year (2023 vs 2022). Marginal effects for each racial and ethnic minority group compared with non-Hispanic White adolescents were estimated at observed values of covariates with non-Hispanic White as the reference group. Marginal effects are presented with 95% CIs and were assessed using 2-tailed tests.

The threshold for statistical significance was *P* < .05 using 2-sided tests. All analyses were conducted with R statistical software version 4.3.2 (R Project for Statistical Computing) and accounted for sampling weights provided by NSDUH to adjust for differential probability of sampling selection, noncoverage bias, and nonresponse bias.^21^

## Results

### Sample Characteristics

Our overall analytic sample included 23 541 adolescents aged 12 to 17 years (8351 aged 14-15 years [35.1%]; 12 167 male [weighted percentage = 51.1%]; 6057 Hispanic [weighted percentage = 26.0%], 1202 non-Hispanic Asian, Hawaiian, or Other Pacific Islander [weighted percentage = 6.2%], 3239 non-Hispanic Black [weighted percentage = 13.8%], 10 756 non-Hispanic White [weighted percentage = 49.7%], and 2287 other non-Hispanic race or ethnicity [weighted percentage = 4.3%]), of whom 5994 adolescents had experienced major depressive episodes in their lifetime. Sample characteristics for the overall sample and the subsample with prior major depressive episodes are presented in Table 1 and eTable 2 in Supplement 1.

**Table 1.** Overall Study Population Characteristics

Characteristic	Total		Asian, Hawaiian, or Other Pacific Islander adolescents^a^			Black adolescents^a^			Hispanic adolescents^a^			White^a^ adolescents		Other adolescents^a^		
	UW No.	W %	UW No.	W %	*P*	UW No.	W %	*P*	UW No.	W %	*P*	UW No.	W %	UW No.	W %	*P*
Adolescents, No.	23 541	NA	1202	NA	NA	3239	NA	NA	6057	NA	NA	10 756	NA	2287	NA	NA
Predisposing characteristics																
Age, y																
12-13	7575	32.0	376	32.7	.99	1032	34.0	.34	1886	29.1	.001	3476	32.7	805	35.0	.25
14-15	8351	35.1	435	36.6	.57	1131	34.6	.68	2185	34.5	.59	3781	35.2	819	36.5	.51
16-17	7615	32.9	391	30.7	.48	1076	31.3	.59	1986	36.4	.004	3499	32.1	663	28.5	.06
Sex																
Male	12 167	51.1	629	52.0	.84	1649	50.2	.42	3093	50.6	.56	5572	51.5	1224	52.4	.65
Female	11 374	48.9	573	48.0	.84	1590	49.8	.42	2964	49.4	.56	5184	48.5	1063	47.6	.65
Household structure																
Mother and father in household	15 616	69.2	1021	87.6	<.001	1238	39.9	<.001	3984	68.0	<.001	8044	76.2	1329	63.2	<.001
Mother or father in household	6720	25.7	155	11.3	<.001	1725	51.4	<.001	1764	26.9	<.001	2286	19.4	790	29.7	<.001
Other or unknown	1205	5.1	26	1.2	<.001	276	8.6	<.001	309	5.2	.21	426	4.4	168	7.1	.01
Enabling characteristics																
Annual family income, $																
<20 000	3411	13.0	105	7.6	.47	946	28.0	<.001	1275	18.9	<.001	733	6.6	352	12.5	<.001
20 000-49 999	6106	24.3	191	16.3	.86	1185	34.5	<.001	2287	35.9	<.001	1822	16.6	621	21.9	.002
50 000-74 999	3250	13.2	165	12.9	.93	409	13.2	.67	860	13.7	.34	1452	12.7	364	16.0	.06
≥75 000	10 774	49.4	741	63.3	.81	699	24.3	<.001	1635	31.5	<.001	6749	64.1	950	49.7	<.001
Insurance status																
Any private	12 166	53.8	819	68.5	.47	1007	34.0	<.001	2017	37.1	<.001	7258	66.2	1065	53.0	<.001
Medicaid (no private)	9646	38.2	312	26.2	.98	2049	59.8	<.001	3459	52.5	<.001	2790	26.3	1036	38.3	<.001
Other (no Medicaid or private)	895	3.6	37	2.7	.09	94	3.3	.22	187	2.8	.008	463	4.1	114	5.4	.20
Uninsured	834	4.4	34	2.6	.41	89	3.0	.39	394	7.7	<.001	245	3.4	72	3.3	.95
English proficiency																
Speaks very well	20 802	88.9	985	83.5	<.001	2946	90.9	.009	4823	80.8	<.001	10 010	93.1	2038	89.7	.01
Speaks not very well	2495	10.1	199	15.4	<.001	237	7.5	.09	1168	17.8	<.001	676	6.2	215	8.7	.05
Unknown or missing	244	1.1	18	1.2	.34	56	1.6	.02	66	1.4	.04	70	0.7	34	1.6	.09
Metropolitan area																
Large	11 086	56.2	772	76.7	<.001	1832	63.6	<.001	3534	63.7	<.001	4084	48.1	864	52.0	.06
Small	8994	31.7	376	20.8	<.001	1085	28.5	.001	1983	29.9	.002	4555	34.6	995	35.9	.54
Nonmetropolitan area	3461	12.0	54	2.5	<.001	322	8.0	<.001	540	6.4	<.001	2117	17.3	428	12.0	<.001
Need-related characteristics																
Major depressive episode in lifetime																
No	16 749	71.7	926	76.3	.004	2472	77.1	<.001	4216	70.0	.50	7562	70.9	1573	67.8	.05
Yes	5994	25.0	235	20.3	<.001	650	19.6	<.001	1602	25.9	.71	2881	26.3	626	28.2	.25
Unknown or missing	798	3.3	41	3.4	.52	117	3.3	.28	239	4.2	.02	313	2.8	88	4.0	.21
Self-rated health																
Fair or poor	1660	6.3	65	5.1	.47	240	6.4	.62	486	6.6	.39	667	6.0	202	8.8	.02
Good, very good, or excellent	21 856	93.6	1136	94.7	.53	2997	93.6	.70	5562	93.3	.42	10 078	93.9	2083	91.2	.03
Self-reported chronic conditions																
None	19 822	85.2	1030	87.5	.28	2613	82.3	.002	5109	85.3	.53	9204	86.0	1866	80.6	.004
≥1	3719	14.8	172	12.5	.28	626	17.7	.002	948	14.7	.53	1552	14.0	421	19.4	.004
Past-year substance use disorder																
No	21 260	91.4	1147	96.2	<.001	2926	91.6	.89	5399	89.9	.03	9777	91.7	2011	89.4	.07
Yes	2281	8.6	55	3.8	<.001	313	8.4	.89	658	10.1	.03	979	8.3	276	10.6	.07

Abbreviations: NA, not applicable; UW, unweighted; W, weighted.

### Racial and Ethnic Differences in Types of Mental Health Service Use

**Figure 1.**
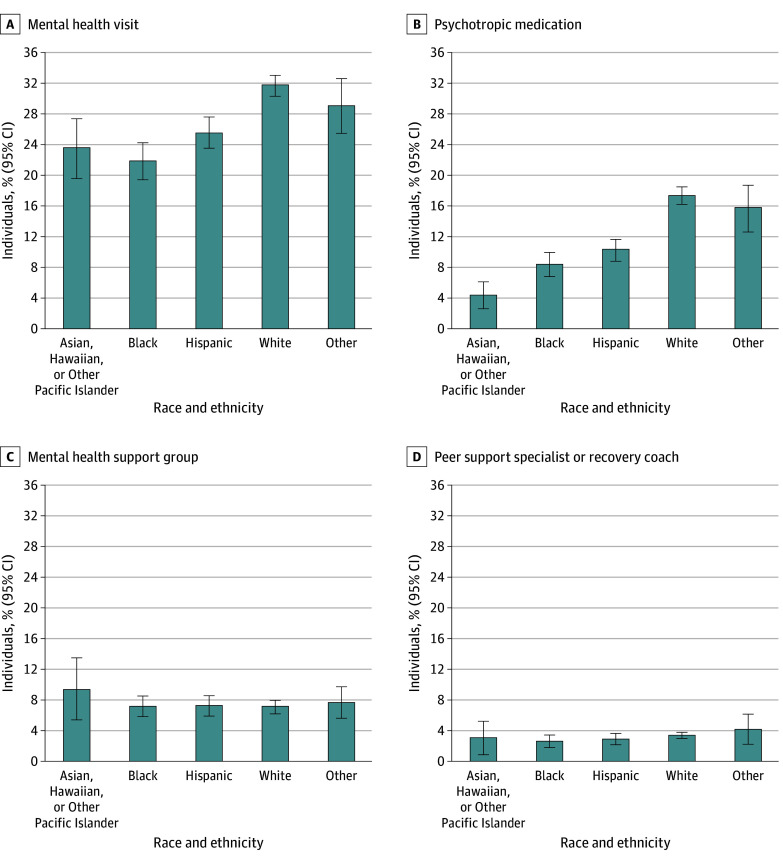
Adjusted Racial and Ethnic Differences in Past-Year Mental Health Service Use in All Adolescents by Treatment Type The analysis was conducted among all adolescents regardless of whether they had a past major depressive episode. Adjusted probabilities for each race and ethnicity group were estimated using weighted logistic regression models and evaluated at the observed values of covariates, with race and ethnicity set as the corresponding race and ethnicity category. Separate logistic regression models were estimated for each treatment type. All estimates are presented with 95% CIs. All groups other than Hispanic were non-Hispanic populations. The other non-Hispanic race and ethnicity group comprised non-Hispanic adolescents who reported more than 1 racial background, as well as Native American or Alaska Native adolescents.

In the overall sample, 6831 adolescents (28.2%) had any mental health visit in the past year (Table 2), but members of racial and ethnic minority groups were significantly less likely to have any mental health visit compared with non-Hispanic White adolescents in unadjusted (Table 2) and adjusted (Table 3; eTable 3 in Supplement 1) comparisons. After adjusting for confounders, Hispanic (−6.1 percentage points [95% CI, −8.7 to −3.6 percentage points]); non-Hispanic Asian, Hawaiian, or Other Pacific Islander (−8.2 percentage points [95% CI, −12.4 to −4.0 percentage points]); and non-Hispanic Black (−9.9 percentage points [95% CI, −12.6 to −7.2 percentage points]) adolescents were less likely (all comparisons, *P* < .001) to have had any mental health visit compared with non-Hispanic White adolescents (Figure 1 and Table 3). Specifically, the adjusted percentage of adolescents receiving any mental health visit was 31.7% (95% CI 30.4%-33.1%) among non-Hispanic White adolescents and was significantly lower among members of racial and ethnic minority groups, ranging from 21.9% (95% CI, 19.5% to 24.3%) among non-Hispanic Black adolescents to 25.6% (95% CI, 23.6% to 27.6%) among Hispanic adolescents.

**Table 2.** Unadjusted Past-Year Mental Health Service Use

Mental health treatment	Total adolescents		Asian, Hawaiian, or Other Pacific Islander adolescents^a^			Black adolescents^a^			Hispanic adolescents			White adolescents^a^		Other adolescents^a^		
	UW No.	W %^b^	UW No.	W %^b^	*P* ^c^	UW No.	W %^b^	*P* ^c^	UW No.	W %^b^	*P* ^c^	UW No.	W %^b^	UW No.	W %^b^	*P* ^c^
**All adolescents**																
No.	23 541	NA	1202	NA	NA	3239	NA	NA	6057	NA	NA	10 756	NA	2287	NA	NA
By type																
Mental health visit	6831	28.2	239	22.0	<.001	715	21.4	<.001	1607	25.2	<.001	3527	32.1	743	31.4	.75
Psychotropic medication	3253	13.6	53	3.9	<.001	277	7.9	<.001	607	10.2	<.001	1949	17.8	367	17.4	.83
Mental health support group	1795	7.4	79	8.4	.44	257	7.8	.29	478	7.6	.45	776	6.9	205	8.6	.23
Peer support specialist or recovery coach	767	3.2	25	2.5	.29	103	2.7	.10	204	3.0	.37	356	3.4	79	5.0	.17
By setting																
Outpatient (clinical)	4394	18.3	130	11.5	<.001	389	12.0	<.001	985	16.5	<.001	2431	21.7	459	19.1	.15
School	3142	12.9	130	11.5	.10	340	10.2	<.001	774	11.6	.008	1544	14.2	354	16.6	.16
Telehealth	3383	14.0	87	7.7	<.001	285	7.9	<.001	728	11.7	<.001	1929	17.5	354	17.0	.80
Inpatient or residential	886	3.2	21	1.5	.03	156	4.0	.06	234	3.3	.63	346	3.0	129	4.8	.10
Emergency department	708	2.7	13	1.7	.20	107	3.1	.44	185	2.5	.66	308	2.7	95	4.6	.10
**With past major depressive episode**																
No.	5994	NA	235	NA	NA	650	NA	NA	1602	NA	NA	2881	NA	626	NA	NA
By type																
Mental health visit	3161	51.3	101	42.4	.02	267	37.9	<.001	747	45.4	<.001	1723	58.0	323	51.0	.15
Psychotropic medication	1662	27.5	33	12.0	<.001	109	14.3	<.001	312	20.5	<.001	1037	35.0	171	30.4	.26
Mental health support group	849	7.9	35	6.0	.38	88	14.1	.06	223	8.1	.99	413	8.1	90	11.5	.32
Peer support specialist or recovery coach	465	13.8	16	16.9	.43	44	5.4	.34	133	14.5	.68	228	13.6	44	12.5	.61
By setting																
Outpatient (clinical)	2184	35.7	61	22.3	<.001	152	21.9	<.001	487	31.7	<.001	1253	41.9	231	34.9	.11
School	1632	26.8	59	27.4	.78	148	18.7	<.001	414	25.5	.20	834	29.0	177	28.2	.82
Telehealth	1901	30.7	56	22.0	.002	135	17.7	<.001	426	25.0	<.001	1094	37.0	190	33.2	.44
Inpatient or residential	408	6.2	8	5.9	.84	48	5.8	.65	103	5.6	.48	196	6.5	53	8.0	.61
Emergency department	432	6.7	5	5.1	.59	53	7.4	.71	103	6.2	.66	210	6.7	61	10.4	.24

Abbreviations: NA, not applicable; UW, unweighted; W, weighted.

^a^ All groups other than Hispanic were non-Hispanic populations. The other non-Hispanic race or ethnicity group comprised non-Hispanic adolescents who reported more than 1 racial background, as well as Native American or Alaska Native adolescents.

^b^ Weighted percentages are presented using the survey package in R statistical software.

^c^ Weighted Wald tests were conducted to compare adolescents in each racial and ethnic minority group with non-Hispanic White adolescents.

**Table 3.** Adjusted Racial and Ethnic Differences in Past-Year Mental Health Service Use

Mental health treatment	Adjusted probability for White, % (95% CI)^a^^,^^b^	Adjusted probability difference vs White, % (95% CI)^a^			
		Asian, Hawaiian, or Other Pacific Islander^b^	Black^b^	Hispanic	Other^b^^,^^c^
**All adolescents**					
By type					
Mental health visit	31.7 (30.4 to 33.1)	−8.2 (−12.4 to −4.0)	−9.9 (−12.6 to −7.2)	−6.1 (−8.7 to −3.6)	−2.6 (−6.5 to 1.3)
Psychotropic medication	17.4 (16.2 to 18.6)	−13.0 (−15.2 to −10.9)	−9.0 (−11.0 to −7.0)	−7.1 (−8.7 to −5.5)	−1.6 (−4.8 to 1.6)
Mental health support group	7.2 (6.4 to 8.0)	2.3 (−1.9 to 6.4)	0.0 (−1.7 to 1.7)	0.1 (−1.5 to 1.7)	0.6 (−1.8 to 3.0)
Peer support specialist or recovery coach	3.4 (3.0 to 3.9)	−0.3 (−2.5 to 1.9)	−0.8 (−1.8 to 0.1)	−0.5 (−1.4 to 0.4)	0.8 (−1.1 to 2.8)
By setting					
Outpatient (clinical)	21.0 (19.8 to 22.1)	−8.7 (−11.1 to −6.3)	−8.1 (−10.6 to −5.7)	−3.7 (−5.8 to −1.7)	−3.5 (−6.8 to −0.2)
School	14.3 (13.2 to 15.4)	−2.2 (−5.6 to 1.1)	−4.0 (−5.9 to −2.0)	−2.8 (−4.5 to −1.0)	1.0 (−2.1 to 4.2)
Telehealth	17.0 (16.0 to 18.0)	−8.9 (−11.8 to −6.0)	−8.4 (−10.5 to −6.4)	−5.0 (−7.0 to −3.0)	−1.5 (−5.0 to 1.9)
Inpatient or residential	3.2 (2.7 to 3.7)	−1.3 (−2.9 to 0.2)	0.6 (−0.4 to 1.6)	−0.2 (−1.2 to 0.8)	1.0 (−0.8 to 2.9)
Emergency department	2.8 (2.3 to 3.3)	−0.7 (−2.4 to 1.0)	0.4 (−1.0 to 1.7)	−0.6 (−1.6 to 0.5)	1.2 (−0.7 to 3.0)
**With past major depressive episode**					
By type					
Mental health visit	57.9 (55.0 to 60.8)	−16.3 (−29.2 to −3.3)	−20.7 (−26.8 to −14.6)	−11.6 (−17.5 to −5.8)	−8.1 (−17.7 to 1.4)
Psychotropic medication	34.8 (32.0 to 37.6)	−22.6 (−29.1 to −16.0)	−20.1 (−24.3 to −15.9)	−13.9 (−18.2 to −9.5)	−5.8 (−13.4 to 1.7)
Mental health support group	14.3 (12.4 to 16.3)	3.1 (−5.3 to 11.4)	−3.9 (−7.5 to −0.2)	−0.6 (−4.5 to 3.4)	−2.3 (−6.3 to 1.7)
Peer support specialist or recovery coach	8.1 (6.7 to 9.6)	−1.0 (−6.9 to 4.8)	−3.1 (−5.8 to −0.4)	0.1 (−2.7 to 2.8)	2.3 (−4.2 to 8.7)
By setting					
Outpatient (clinical)	41.1 (38.2 to 44.0)	−19.8 (−28.9 to −10.8)	−18.5 (−24.9 to −12.1)	−7.8 (−12.7 to −2.9)	−7.5 (−16.2 to 1.2)
School	29.3 (26.8 to 31.9)	−3.2 (−14.1 to 7.7)	−11.6 (−16.2 to −7.0)	−3.5 (−8.6 to 1.5)	−1.6 (−8.4 to 5.2)
Telehealth	36.8 (33.8 to 39.8)	−16.2 (−24.4 to −8.0)	−19.0 (−24.6 to −13.4)	−11.1 (−16.1 to −6.1)	−4.7 (−14.1 to 4.8)
Inpatient or residential	6.6 (5.4 to 7.8)	0.2 (−6.3 to 6.7)	−0.6 (−3.7 to 2.5)	−1.2 (−3.7 to 1.3)	0.6 (−4.6 to 5.8)
Emergency department	7.0 (5.6 to 8.4)	−1.2 (−7.6 to 5.3)	−0.3 (−4.1 to 3.5)	−1.1 (−3.8 to 1.7)	2.6 (−3.2 to 8.5)

^a^ Adjusted probabilities and marginal effects are presented in percentage points. Adjusted probabilities for non-Hispanic White adolescents were estimated using weighted logistic regression models and evaluated at the observed values of covariates, with race and ethnicity set as non-Hispanic White. Marginal effects for each racial and ethnic minority group compared with non-Hispanic White adolescents were estimated using the same model and evaluated at the observed values of covariates, with non-Hispanic White as the reference group. All estimates are presented with 95% CIs and were assessed using 2-tailed tests.

^b^ All groups other than Hispanic were non-Hispanic populations.

^c^ The other non-Hispanic race and ethnicity group comprised non-Hispanic adolescents who reported more than 1 racial background, as well as Native American or Alaska Native adolescents.

Similarly, among all adolescents, 3253 adolescents (13.6%) reported receiving any mental health prescription medication in the past year, both unadjusted (Table 2) and adjusted (Figure 1 and Table 3) percentages were significantly lower among members of racial and ethnic minority groups than non-Hispanic White adolescents. Results from adjusted analyses showed that Hispanic (−7.1 percentage points [95% CI, −8.7 to −5.5 percentage points]); non-Hispanic Asian, Hawaiian, or Other Pacific Islander (−13.0 percentage points [95% CI, −15.2 to −10.9 percentage points]); and non-Hispanic Black (−9.0 percentage points [95% CI, −11.0 to −7.0 percentage points]) adolescents were less likely (all comparisons, *P* < .001) to report receiving any mental health prescription medication than non-Hispanic White adolescents (adjusted percentage = 17.4% [95% CI, 16.2% to 18.6%]) (Figure 1 and Table 3).

Among all adolescents, 1795 adolescents (7.4%) reported participating in a mental health support group and 767 adolescents (3.2%) reported receiving help from mental health peer support specialists or recovery coaches (Table 2). There were no significant racial or ethnic differences in the receipt of these services in unadjusted (Table 2) or adjusted comparisons (Figure 1 and Table 3).

In the subsample of adolescents with a past major depressive episode, 3161 adolescents (51.3%) reported having a mental health visit in the past year, while 1662 adolescents (27.5%) reported receiving mental health prescription medication, 849 adolescents (7.9%) reported participating in a mental health support group, and 465 adolescents (13.8%) reported receiving help from mental health peer support specialists or recovery coaches in the past year (Table 2). As in the overall sample, members of racial and ethnic minority groups were significantly less likely to have a mental health visit or receive mental health prescription medication compared with non-Hispanic White adolescents in unadjusted (Table 2) and adjusted (Table 3; eTable 4 in Supplement 1) comparisons. Specifically, compared with non-Hispanic White adolescents (adjusted percentage = 57.9% [95% CI, 55.0%-60.8%]), the adjusted difference in having any mental health visits ranged from 11.6 percentage points (95% CI, 5.8-17.5 percentage points) lower among Hispanic adolescents to 20.7 percentage points (95% CI, 14.6-26.8 percentage points) lower among non-Hispanic Black adolescents (Table 3; eTable 4 and eFigure 1 in Supplement 1). Similarly, compared with non-Hispanic White adolescents (adjusted percentage = 34.8% [95% CI, 32.0%-37.6%]), the adjusted difference in receiving mental health prescription medication ranged from 13.9 percentage points (95% CI, 9.5-18.2 percentage points) lower among Hispanic adolescents to 22.6 percentage points (95% CI, 16.0-29.1 percentage points) lower among non-Hispanic Asian, Hawaiian, or Other Pacific Islander adolescents (Table 3; eTable 4 and eFigure 1 in Supplement 1).

### Racial and Ethnic Differences in Mental Health Service Use by Setting

**Figure 2.**
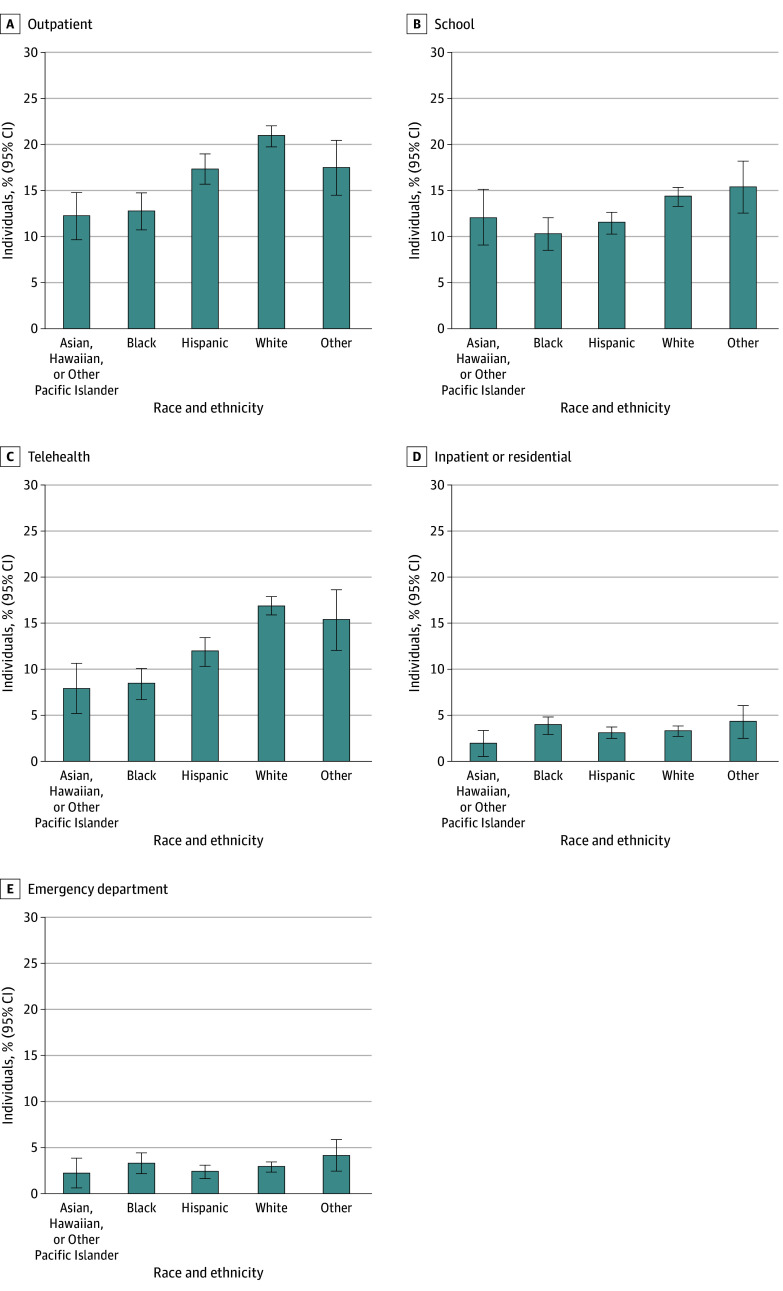
Adjusted Racial and Ethnic Differences in Past-Year Mental Health Service Use in All Adolescents by Treatment Setting The analysis was conducted among all adolescents regardless of whether they had a past major depressive episode. Adjusted probabilities for each race and ethnicity group were estimated using weighted logistic regression models and evaluated at the observed values of covariates, with race and ethnicity set as the corresponding race and ethnicity category. Separate logistic regression models were estimated for each treatment setting. All estimates are presented with 95% CIs. All groups other than Hispanic were non-Hispanic populations. The other non-Hispanic race and ethnicity group comprised non-Hispanic adolescents who reported more than 1 racial background, as well as Native American or Alaska Native adolescents.

In the overall sample, 4394 adolescents (18.3%) reported receiving any mental health services in a clinical outpatient setting, and there were significant racial and ethnic differences observed in this outcome in unadjusted (Table 2) and adjusted (Table 3; eTable 5 in Supplement 1) comparisons. After adjusting for confounding variables, Hispanic (−3.7 percentage points [95% CI, −5.8 to −1.7 percentage points]); non-Hispanic Asian, Hawaiian, or Other Pacific Islander (−8.7 percentage points [95% CI, −11.1 to −6.3 percentage points]); and non-Hispanic Black (−8.1 percentage points [95% CI, −10.6 to −5.7 percentage points]) adolescents were less likely (all comparisons *P* < .001) to have received outpatient mental health treatment compared with non-Hispanic White adolescents (adjusted percentage = 21.0% [95% CI, 19.8% to 22.1%]) (Figure 2 and Table 3).

Telemental health was the next most common setting in which services were received by adolescents during the year (3383 adolescents [14.0%]), and there were also significant racial and ethnic differences in this outcome (Table 2 and Table 3; eTable 5 in Supplement 1). Compared with non-Hispanic White adolescents (adjusted percentage = 17.0% [95% CI, 16.0%-18.0%]), adjusted differences ranged from 5.0 percentage points (95% CI, 3.0-7.0 percentage points) lower among Hispanic adolescents to 8.9 percentage points (95% CI, 6.0-11.8 percentage points) lower among non-Hispanic Asian, Hawaiian, or Other Pacific Islander adolescents. Among adolescents who were members of racial and ethnic minority groups, the percentage ranged from 8.1% (95% CI, 5.4%-10.8%) among non-Hispanic Asian, Hawaiian, or Other Pacific Islander adolescents to 12.0% (95% CI, 10.4%-13.6%) among Hispanic adolescents (all *P* < .001).

Racial and ethnic differences were also observed in mental health services received in school settings. Overall, 3142 adolescents (12.9%) received any mental health service in the school setting, and significant racial and ethnic disparities were observed comparing non-Hispanic White adolescents (1544 adolescents [14.2%]) with Hispanic (774 adolescents [11.6%]; *P* = .008) and non-Hispanic Black (340 adolescents [10.2%]; *P* < .001) adolescents (Table 2). These differences remained significant in regression analysis (non-Hispanic Black: −4.0 percentage points [95% CI, −5.9 to −2.0 percentage points]; *P* < .001; Hispanic: −2.8 percentage points [95% CI, −4.5 to −1.0 percentage points]; *P* = .002) (Figure 2 and Table 3).

There were few racial or ethnic differences observed in mental health services received in inpatient or residential settings or the emergency department (Table 2 and Table 3; eTable 5 in Supplement 1), with 886 adolescents (3.2%) and 708 adolescents (2.7%) reporting receiving any mental health treatment in each setting, respectively (Table 2). Compared with non-Hispanic White adolescents (346 adolescents [3.0%]), non-Hispanic Asian, Hawaiian, or Other Pacific Islander adolescents were less likely to receive mental health treatment in an inpatient or a residential setting (21 adolescents [1.5%]; *P* = .03) in unadjusted comparisons (Table 2). This difference was no longer significant in the regression model (Table 3). Finally, there were no racial or ethnic differences observed in mental health service use in the emergency department in bivariate or multivariate comparisons.

In the subsample of adolescents with a past major depressive episode, 2184 adolescents (35.7%) reported receiving mental health services in a clinical outpatient setting, 1901 adolescents (30.7%) reported in telemental health setting, 1632 adolescents (26.8%) reported in school settings, 432 adolescents (6.7%) reported in emergency departments, and 408 adolescents (6.2%) reported in inpatient or residential settings (Table 2). Similar to findings in the overall sample, Hispanic; non-Hispanic Asian, Hawaiian, or Other Pacific Islander; and non-Hispanic Black adolescents with a prior episode of major depression were significantly less likely to receive mental health services in clinical outpatient or telemental health settings in unadjusted and adjusted comparisons (Table 2 and Table 3; eTable 6 in Supplement 1). Unlike in the overall sample, significant racial and ethnic differences in school settings were observed only between non-Hispanic White and non-Hispanic Black adolescents (adjusted difference, −11.6 percentage points [95% CI, −16.2 to −7.0 percentage points) but not between non-Hispanic White and Hispanic adolescents (Table 3; eTable 6 and eFigure 2 in Supplement 1).

## Discussion

This cross-sectional study provides crucial, contemporary data on racial and ethnic differences in adolescent mental health service use from a national survey. During the study period, 28.2% of US adolescents had some form of mental health visit, yet large and significant racial and ethnic differences were observed in the receipt of any mental health visit, outpatient treatment in a clinical setting, mental health services in a school setting, telemental health treatment, and psychotropic medication. Conversely, there were smaller to no racial and ethnic differences observed in the receipt of mental health services from support groups, peer support specialists and recovery coaches, inpatient and residential settings, or emergency departments. Similar patterns were observed among adolescents with prior major depressive episodes.

Consistent with prior literature, we found large and significant racial and ethnic differences in the receipt of any mental health service, clinical outpatient services, and telemental health services.^9,22^ Furthermore, given that these are the first estimates from a large, national survey, our study adds to an emerging evidence base about racial and ethnic differences in child and adolescent telemental health^18,23^ by documenting the large and significant racial and ethnic differences in telemental health treatment among adolescents, the second most common service setting between 2022 and 2023. Stigma, mistrust, and historical experiences of discrimination may make adolescent members of racial and ethnic minority groups and their families hesitant or reluctant to engage with mental health services.^24,25,26^ The absence of physical interaction, which typically helps build patient-clinician rapport, in telemental health visits may create further distance between the family and clinician and therefore amplify mistrust among racial and ethnic minority groups.^27,28^ Future research is needed to understand whether racial and ethnic differences in trust toward the mental health system contribute to differences in telemental health use, as well as the extent to which disparities in telemental health use may exacerbate racial and ethnic differences in adolescent access to the mental health system.

Psychotropic medication use was found to be significantly lower among all adolescent members of racial and ethnic minority groups, adding to the current evidence base.^10,29,30,31^ Recent research using the National Survey of Children’s Health and Medical Expenditure Panel Survey found that child and youth members of racial and ethnic minority groups were less likely to use psychotropic medication overall and among those with mental or behavioral health problems.^10,29,30,31^ Notably, Cook et al^31^ found that these differences were likely driven by overprescribing among White youths, as well as underprescribing among youths in minority racial and ethnic populations with mental health needs.

We found that non-Hispanic Black adolescents were significantly less likely than non-Hispanic White adolescents to report receiving school-based mental health services, both overall and among those with prior major depressive episodes. However, pre–COVID-19 studies found no significant differences between Black and White adolescents in school-based mental health service use among those with major depressive episodes^6,14^ and documented higher use among non-Hispanic Black adolescents overall.^32^ Such discrepancies may reflect changes in the school mental health services measure in the 2022 to 2023 NSDUH. Specifically, the survey’s new skip pattern and question wording likely led to a narrower interpretation of the types of mental health services received in the school setting (ie, more clinical services), compared with more inclusive wording for mental health counseling, services, and programs in prior surveys. This change may have also contributed to the lower reported school-based mental health use in 2022 to 2023 (12.9%) compared with 2019 (15.7%).^32^

While we observed large racial and ethnic differences for many outcomes, it is notable that there were no racial or ethnic differences in receiving mental health services from support groups or peer support specialists and recovery coaches. Previous literature highlights the benefits for adolescents in seeking mental health services from peer support specialists, including that these specialists provide emotional support, advocate for patient needs, and assist in coordinating care.^33,34,35^ Moreover, the availability of peer support has been associated with reduced differences in annual outpatient mental health service use in Latino and Black youths compared with non-Latino White youths in local samples.^34^ Enhancing culturally relevant, community-based group and peer services tailored specifically to adolescents in racial and ethnic minority groups may offer an approach to support minoritized youth populations as they seek services.

### Limitations

While this study provides valuable recent data on existing racial and ethnic differences in the setting in which adolescents receive mental health services, several limitations should be acknowledged. First, given the observational, cross-sectional design of this study, we can make no conclusions regarding causal relationships between racial and ethnic background and mental health service use. Additionally, the measurement of school mental health services in the 2022 to 2023 NSDUH is not directly comparable to those from previous years due to changes in question wording. In another limitation, mental health use measures in this study were based on self-report and thus susceptible to misclassification. Nonetheless, most of the NSDUH interview was conducted by audio, computer-assisted self-interviewing technology, designed to reduce social desirability bias and other response biases related to sensitive topics.^21^

## Conclusions

This cross-sectional study documents important racial and ethnic differences in adolescent mental health service use after the COVID-19 pandemic using a national database. We found substantial racial and ethnic differences in psychotropic medication and mental health service use in clinical outpatient, school, and telemental health settings. These findings highlight the need to improve mental health access for adolescents in minority racial and ethnic groups.

## Notes

Supplement 1. **eAppendix 1.** 2022-2023 National Survey on Drug Use and Health Questions on Mental Health Services Utilization **eAppendix 2.** 2022-2023 National Survey on Drug Use and Health Questions on Major Depressive Episode **eTable 1.** Sociodemographic and Health Status Measures **eTable 2.** Sample characteristics of adolescents (age 12 to 17) with past major depressive episode in 2022-2023, by race/ethnicity **eTable 3.** Racial/ethnic differences in past-year mental health service use among all adolescents, by treatment type **eTable 4.** Racial/ethnic differences in past-year mental health service use among adolescents with past major depressive disorder, by treatment type **eTable 5.** Racial/ethnic differences in past-year mental health service use among all adolescents, by treatment setting **eTable 6.** Racial/ethnic differences in past-year mental health service use among adolescents with past major depressive episodes, by treatment setting **eFigure 1.** Adjusted racial/ethnic differences in past-year mental health service use among adolescents with past major depressive disorder, by treatment type, 2022-2023 **eFigure 2.** Adjusted racial/ethnic differences in past-year mental health service use among adolescents with past major depressive disorder, by treatment setting, 2022-2023

Supplement 2.
Data Sharing Statement
