# Supplementary material for: Racial and Ethnic Differences in Mental Health Service Use Among Adolescents
Source: JAMA Netw Open. 2025 Jun 18;8(6):e2516612. doi: 10.1001/jamanetworkopen.2025.16612 (PMC12177673; doi:10.1001/jamanetworkopen.2025.16612)
Supplement: Supplement 1. — eAppendix 1. 2022-2023 National Survey on Drug Use and Health Questions on Mental Health Services Utilization eAppendix 2. 2022-2023 National Survey on Drug Use and Health Questions on Major Depressive Episode eTable 1. Sociodemographic and Health Status Measures eTable 2. Sample characteristics of adolescents (age 12 to 17) with past major depressive episode in 2022-2023, by race/ethnicity eTable 3. Racial/ethnic differences in past-year mental health service use among all adolescents, by treatment type eTable 4. Racial/ethnic differences in past-year mental health service use among adolescents with past major depressive disorder, by treatment type eTable 5. Racial/ethnic differences in past-year mental health service use among all adolescents, by treatment setting eTable 6. Racial/ethnic differences in past-year mental health service use among adolescents with past major depressive episodes, by treatment setting eFigure 1. Adjusted racial/ethnic differences in past-year mental health service use among adolescents with past major depressive disorder, by treatment type, 2022-2023 eFigure 2. Adjusted racial/ethnic differences in past-year mental health service use among adolescents with past major depressive disorder, by treatment setting, 2022-2023 [file jamanetwopen-e2516612-s001.pdf]

Supplemental Online Content

Ma Y, Ramos C, Wen H, Cummings JR. Racial and ethnic differences in mental health service use among adolescents. *JAMA Netw Open*. 2025;8(6):e2516612. doi:10.1001/jamanetworkopen.2025.16612

eAppendix 1. 2022-2023 National Survey on Drug Use and Health Questions on Mental Health Services Utilization

eAppendix 2. 2022-2023 National Survey on Drug Use and Health Questions on Major Depressive Episode

eTable 1. Sociodemographic and Health Status Measures

eTable 2. Sample characteristics of adolescents (age 12 to 17) with past major depressive episode in 2022-2023, by race/ethnicity

eTable 3. Racial/ethnic differences in past-year mental health service use among all adolescents, by treatment type

eTable 4. Racial/ethnic differences in past-year mental health service use among adolescents with past major depressive disorder, by treatment type

eTable 5. Racial/ethnic differences in past-year mental health service use among all adolescents, by treatment setting

eTable 6. Racial/ethnic differences in past-year mental health service use among adolescents with past major depressive episodes, by treatment setting

eFigure 1. Adjusted racial/ethnic differences in past-year mental health service use among adolescents with past major depressive disorder, by treatment type, 2022-2023

eFigure 2. Adjusted racial/ethnic differences in past-year mental health service use among adolescents with past major depressive disorder, by treatment setting, 2022-2023

This supplemental material has been provided by the authors to give readers additional information about their work.

© 2025 Ma Y et al. *JAMA Network Open*.

eAppendix 1 2022-2023 National Survey on Drug Use and Health Questions on Mental Health Services Utilization

During the past 12 months, have you participated in a **support group** to help you with your mental health, emotions, or behavior?

1 Yes

2 No

During the past 12 months, were you seen in an **emergency room or emergency department** for your mental health, emotions, or behavior?

1 Yes

2 No

The next questions ask about professional counseling, medication, or other treatment you may have received for your mental health, emotions, or behavior where you **stayed overnight or longer**. This is sometimes called inpatient or residential treatment.

During the past 12 months, have you **stayed overnight or longer** to receive professional counseling, medication, or other treatment for your mental health, emotions, or behavior in…

**Stayed overnight or longer for treatment in…**	**Yes**	**No**
a hospital as an inpatient?	1	2
a residential mental health treatment center?	1	2
a residential drug or alcohol rehab or treatment center?	1	2
some other place where you stayed overnight or longer?	1	2

Professional counseling, medication or other treatment can also be provided **without** needing to stay overnight. This type of care is called **outpatient** treatment.

During the past 12 months, have you received **outpatient** professional counseling, medication, or other treatment for your mental health, emotions, or behavior at…

**Received outpatient treatment at…**	**Yes**	**No**
a mental health treatment center as an outpatient?	1	2
a drug or alcohol treatment or rehab center as an outpatient?	1	2
the office of a therapist, psychologist, psychiatrist, or mental health professional?	1	2
a general medical clinic or doctor's office?	1	2
a hospital as an outpatient?	1	2
your school's health or counseling center?	1	2
some other place as an outpatient?	1	2

© 2025 Ma Y et al. *JAMA Network Open*.

During the past 12 months, did you take any **medication** that was prescribed to you to help with your mental health, emotions, or behavior?

1 Yes

2 No

During the past 12 months have you received any professional counseling, medication or treatment for your mental health, emotions, or behavior from a therapist or other healthcare professional **over the phone, or through video**?

1 Yes

2 No

During the past 12 months, have you received help from **a peer support specialist or recovery coach** who works with a mental health treatment program or other treatment provider?

1 Yes

2 No

© 2025 Ma Y et al. *JAMA Network Open*.

eAppendix 2 2022-2023 National Survey on Drug Use and Health Questions on Major Depressive Episode

NSDUH measures the nine symptoms associated with MDE as defined in DSM-5 with the following questions for youths aged 12 to 17.

In answering the next questions, think about the period of time when your [FEELNOUN] and other problems were the **worst/most recent**

1. **Depressed mood most of the day**

    a. During that time, did you feel sad, empty, or depressed for **most of the day nearly every day**?
    b. During that [TIMEFILL] period of time, did you feel discouraged about how things were going in your life **most of the day nearly every day**?

2. Markedly diminished interest or pleasure in all or almost all activities most of the day

    a. During that [TIMEFILL] period of time, did you become bored with almost everything like school, work, hobbies, and things you like to do for fun?
    b. During that [TIMEFILL] period of time, did you feel like nothing was fun even when good things were happening?

3. **Weight**

The next questions are about changes in appetite and weight.

a. Did you eat much less than usual almost every day during that time?
b. Did you eat much more than usual almost every day?
c. Did you gain weight without trying to during that [TIMEFILL] period of time?
    Did you gain weight without trying to because you were growing?
    Did you gain weight without trying to because you were pregnant?
    How many pounds did you gain?
d. Did you lose weight without trying to?
    Did you lose weight without trying to because you were sick or on a diet?
    How many pounds did you lose?

4. **Insomnia or hypersomnia**

    a. Did you have a lot more trouble than usual falling asleep or staying asleep most nights or waking too early most mornings during that [TIMEFILL] time?
    b. During that [TIMEFILL] period of time, did you sleep a lot more than usual?

5. **Psychomotor agitation or retardation**

© 2025 Ma Y et al. *JAMA Network Open*.

a. Did you feel as though you were talking or moving more slowly than usual on most days during that [TIMEFILL] period of time?
b. Did anyone else notice that you were talking or moving more slowly than usual?
c. Were you so restless or jittery that you walked up or down or couldn't sit still?
d. Did anyone else notice that you couldn't sit still?

6. **Fatigue or loss of energy**

a. On most days during that [TIMEFILL] period of time, did you feel that you didn't have much energy?

7. **Feelings of worthlessness**

a. Did you feel that you were not as good as other people nearly every day?
b. Did you feel totally worthless nearly every day?

8. **Diminished ability to think or concentrate or indecisiveness**

a. On most days during that [TIMEFILL] time, did your thinking seem slower than usual or seem mixed up?
b. On most days, did you have a lot more trouble than usual keeping your mind on things?
c. Were you unable to make up your mind about things you ordinarily have no trouble deciding about?

9. **Recurrent thoughts of death or recurrent suicidal ideation**

a. Did you often think a lot about death, either your own, someone else's, or death in general?
b. During that time, did you ever think that it would be better if you were dead?
c. Did you think about killing yourself?
   Did you make a plan to kill yourself?
   Did you make a suicide attempt or try to kill yourself?

© 2025 Ma Y et al. *JAMA Network Open*.

eTable 1 Sociodemographic and Health Status Measures

Measure	Type	Value
Predisposing Characteristics		
Age	categorical	12–13,<br>14-15,<br>16-17
Sex	binary	Male,<br>Female
Household Structure	categorical	Mother and father in household,<br>Mother or father in household,<br>Other or unknown
Enabling Characteristics		
Family Income	categorical	<$20,000,<br>$20,000 - $49,999,<br>$50,000 - $74,999,<br>$75,000 or more
Insurance Status	categorical	Any private insurance,<br>Medicaid (no private insurance),<br>Other Insurance (no private, no Medicaid),<br>Uninsured
English Proficiency	categorical	Very well,<br>Not very well,<br>Unknown or missing
Metropolitan Area	categorical	Large metropolitan area,<br>Small metropolitan area,<br>Non-metropolitan area
Need-related Characteristics		
Major depressive episode	categorical	Any episode in lifetime<br>No episode in lifetime<br>Unknown or missing
Self-rated health	binary	Fair / poor,<br>Good / very good / excellent
Self-reported chronic conditions	binary	None,<br>One or More
Past-year substance use disorder	binary	No<br>Yes

© 2025 Ma Y et al. *JAMA Network Open*.

eTable 2 Sample characteristics of adolescents (age 12 to 17) with past major depressive episode in 2022-2023, by race/ethnicity

	All	Asian, Hawaiian, or other Pacific Islander	Black	Hispanic	White	Other
N	5,994	235	650	1,602	2,881	626
Predisposing Characteristics						
Age, %						
12-13 Years Old	20.8	17.7	27.9*	18.1	20.9	22.6
14-15 Years Old	37.2	41.2	32.5	36.9	38.1	36.1
16-17 Years Old	42	41.1	39.6	45.0	41.1	41.3
Sex, %						
Male	29.9	27.5	27.6	29.9	30.6	28.7
Female	70.1	72.5	72.4	70.1	69.4	71.3
Household Structure, %						
Mother and father in household	68	89.5***	42.6***	64.9**	73.4	61.8*
Mother or father in household	27.4	10.0***	51.1***	30.4**	22.3	31.3*
Other or unknown	4.6	0.5***	6.3	4.7	4.3	6.9
Enabling Characteristics						
Annual Family Income, %						
<$20,000	12	5.1	24.5***	18.1***	7.1	11.2
$20,000 - $49,999	24.2	12.6	29.2***	36.0***	18.3	23.0
$50,000 - $74,999	14.9	11.6	16.6	14.9	14.4	20.5
$75,000 or more	48.9	70.8**	29.7***	31.1***	60.2	45.2***
Insurance Status, %						
Any private insurance	54.8	71.4	42.1***	37.6***	64.7	54.5**
Medicaid (no private insurance)	37.3	26.1	52.3***	53.1***	27.3	35.9*
Other Insurance (no private, no Medicaid)	4.1	1.0***	3.4	2.9	4.9	7.3
Uninsured	3.8	1.4	2.2	6.5*	3.1	2.2
English Proficiency, %						
Very well	88.8	84.8*	93.2	80.4***	92.4	90.7
Not very well	11.1	15.2**	6.8	19.6***	7.4	9.0
Metropolitan Area, %						
Large metropolitan area	57.3	80.5***	73.2***	64.5***	48.5	53.1
Small metropolitan area	31.2	17.0***	19.7***	30.6	34.7	36.1
Non-metropolitan area	11.5	2.5***	7.1***	4.9***	16.8	10.9*
Need-related Characteristics						
Self-rated health , %						
Fair or poor	12.6	13.3	11.6	10.9	13.3	16.9
Good, very good, or excellent	87.3	86.7	88.4	89.1	86.7	83.1
Self-reported chronic conditions, %						
None	79.8	80.1	77.5	79.1	80.8	77.5
One or More	20.2	19.9	22.5	20.9	19.2	22.5
Past-year substance use disorder, %						
No	82.6	91.3***	85.1	81.7	81.9	80.2
Yes	17.4	8.7***	14.9	18.3	18.1	19.8

Notes: * p<0.05, ** p<0.01, *** p<0.001.
Weighted Wald tests were conducted to compare adolescents in each racial/ethnic minority group to non-Hispanic White adolescents.
Weighted percentages are presented using the survey package in R.

© 2025 Ma Y et al. *JAMA Network Open*.

eTable 3 Racial/ethnic differences in past-year mental health service use among all adolescents, by treatment type

	Any Mental Health Visit	Any Psychotropic Medication	Any Mental Health Support Group	Any Peer Support Specialist / Recovery Coach
	Estimate (95% CI)	Estimate (95% CI)	Estimate (95% CI)	Estimate (95% CI)
**Adjusted Probability for White**	31.7***	17.4***	7.2***	3.4***
	(30.4,33.1)	(16.2,18.6)	(6.4,8.0)	(3.0,3.9)
**Marginal Effects**				
Race/Ethnicity				
Asian, Hawaiian, or other Pacific Islander	-8.2***	-13.0***	2.3	-0.3
	(-12.4,-4.0)	(-15.2,-10.9)	(-1.9,6.4)	(-2.5,1.9)
Black	-9.9***	-9.0***	0.0	-0.8
	(-12.6,-7.2)	(-11.0,-7.0)	(-1.7,1.7)	(-1.8,0.1)
Hispanic	-6.1***	-7.1***	0.1	-0.5
	(-8.7,-3.6)	(-8.7,-5.5)	(-1.5,1.7)	(-1.4,0.4)
Other	-2.6	-1.6	0.6	0.8
	(-6.5,1.3)	(-4.8,1.6)	(-1.8,3.0)	(-1.1,2.8)
Predisposing Characteristics				
Age				
12-13 Years Old (Reference)				
14-15 Years Old	-2.5	-0.6	0.0	0.0
	(-5.2,0.3)	(-2.7,1.6)	(-1.3,1.3)	(-1.0,1.1)
16-17 Years Old	-6.5***	-0.4	-2.7***	-1.1*
	(-8.9,-4.1)	(-2.7,1.8)	(-3.7,-1.7)	(-2.2,-0.1)
Sex				
Male (Reference)				
Female	7.9***	0.8	1.7**	1.9***
	(5.9,9.9)	(-1.3,2.9)	(0.6,2.9)	(1.2,2.6)
Household Structure				
Mother or father in household (Reference)				
Mother and father in household	-5.1***	-2.1	-1.0	-1.4*
	(-7.8,-2.4)	(-4.3,0.2)	(-2.4,0.3)	(-2.6,-0.2)
Other or unknown	-4.7*	0	-0.7	-1.1
	(-9.1,-0.4)	(-3.6,3.6)	(-3.1,1.7)	(-2.8,0.7)

© 2025 Ma Y et al. *JAMA Network Open*.

	Any Mental Health Visit	Any Psychotropic Medication	Any Mental Health Support Group	Any Peer Support Specialist / Recovery Coach
	Estimate (95% CI)	Estimate (95% CI)	Estimate (95% CI)	Estimate (95% CI)
Enabling Characteristics				
Family Income				
<$20,000 (Reference)				
$20,000 - $49,999	0.9	0.0	-0.5	1.2*
	(-2.3,4.0)	(-2.7,2.6)	(-2.6,1.5)	(0.1,2.3)
$50,000 - $74,999	4.0	3.3**	-2.3*	0.3
	(-0.2,8.2)	(0.9,5.6)	(-4.6,0.0)	(-1.0,1.7)
$75,000 or more	2.3	3.6**	-2.9**	0.5
	(-1.2,5.9)	(0.9,6.4)	(-5.0,-0.7)	(-0.8,1.8)
Insurance Status				
Any private insurance (Reference)				
Medicaid (no private insurance)	-2.4	0.7	-0.9	0.2
	(-5.0,0.2)	(-1.7,3.2)	(-2.5,0.7)	(-1.0,1.4)
Other insurance (no private, no Medicaid)	-3.7	-5.3**	0.3	0.1
	(-8.3,0.9)	(-8.5,-2.1)	(-3.0,3.6)	(-2.4,2.6)
Uninsured	-10.2***	-9.2***	-3.3**	-0.7
	(-15.4,-5.0)	(-13.3,-5.1)	(-5.4,-1.3)	(-2.7,1.3)
English Proficiency				
Not very well (Reference)				
Very well	0.8	-1.2	0.7	0.3
	(-2.1,3.8)	(-4.1,1.7)	(-1.2,2.5)	(-0.8,1.4)
Unknown/Missing	3.0	0.6	3.3	5.9*
	(-6.1,12.1)	(-6.3,7.5)	(-2.6,9.3)	(0.2,11.6)
Metropolitan Area				
Large metropolitan area (Reference)				
Small metropolitan area	-1.2	0.2	-0.2	0.5
	(-3.0,0.7)	(-1.8,2.3)	(-1.3,0.8)	(-0.3,1.2)
Non-metropolitan area	-3.0	-1.1	-1.3	0.0
	(-6.2,0.1)	(-3.2,1.1)	(-3.2,0.6)	(-0.9,1.0)

© 2025 Ma Y et al. *JAMA Network Open*.

	Any Mental Health Visit	Any Psychotropic Medication	Any Mental Health Support Group	Any Peer Support Specialist / Recovery Coach
	Estimate (95% CI)	Estimate (95% CI)	Estimate (95% CI)	Estimate (95% CI)
Need-related Characteristics				
Major Depressive Episode in life time				
No (Reference)				
Yes	27.4***	18.0***	6.7***	4.7***
	(24.9,29.8)	(15.6,20.4)	(5.3,8.1)	(3.5,5.8)
Unknown/Missing	8.1*	2.0	1.2	-0.2
	(1.5,14.7)	(-2.6,6.5)	(-1.1,3.5)	(-1.3,1.0)
Self-rated Health				
Good, very good, or excellent (Reference)				
Fair or poor	10.5***	9.3***	4.5**	3.1**
	(6.0,15.1)	(5.3,13.3)	(1.7,7.3)	(1.1,5.0)
Any Self-reported Chronic Conditions				
No (Reference)				
Yes	9.2***	5.9***	2.4**	1.2**
	(6.8,11.6)	(3.3,8.5)	(0.7,4.0)	(0.4,2.0)
Past Year Substance Use Disorder				
No (Reference)				
Yes	10.2***	13.1***	4.5***	2.9***
	(6.9,13.6)	(10.3,15.8)	(2.3,6.6)	(1.4,4.3)
Year	1.2	1.2	0.2	-0.1
	(-0.7,3.1)	(-0.9,3.3)	(-0.7,1.0)	(-0.9,0.7)

Notes: * $p<0.05$, ** $p<0.01$, *** $p<0.001$.

Adjusted probabilities and marginal effects were presented in percentage points. Adjusted probabilities for non-Hispanic Whites were estimated using weighted logistic regression models and evaluated at the observed values of the covariates with race/ethnicity set as non-Hispanic Whites. Marginal effects for each racial/ethnic minority group relative to non-Hispanic Whites were estimated using the same model and evaluated at the observed values of the covariates with non-Hispanic White as the reference group. All estimates were presented with 95% confidence intervals and assessed using two-tailed tests.

© 2025 Ma Y et al. *JAMA Network Open*.

eTable 4 Racial/ethnic differences in past-year mental health service use among adolescents with past major depressive disorder, by treatment type

	Any Mental Health Visit	Any Psychotropic Medication	Any Mental Health Support Group	Any Peer Support Specialist / Recovery Coach
	Estimate (95% CI)	Estimate (95% CI)	Estimate (95% CI)	Estimate (95% CI)
**Adjusted Probability for White**	57.9***	34.8***	14.3***	8.1***
	(55.0,60.8)	(32.0,37.6)	(12.4,16.3)	(6.7,9.6)
**Marginal Effects**				
Race/Ethnicity				
Asian, Hawaiian, or other Pacific Islander	-16.3*	-22.6***	3.1	-1.0
	(-29.2,-3.3)	(-29.1,-16.0)	(-5.3,11.4)	(-6.9,4.8)
Black	-20.7***	-20.1***	-3.9*	-3.1*
	(-26.8,-14.6)	(-24.3,-15.9)	(-7.5,-0.2)	(-5.8,-0.4)
Hispanic	-11.6***	-13.9***	-0.6	0.1
	(-17.5,-5.8)	(-18.2,-9.5)	(-4.5,3.4)	(-2.7,2.8)
Other	-8.1	-5.8	-2.3	2.3
	(-17.7,1.4)	(-13.4,1.7)	(-6.3,1.7)	(-4.2,8.7)
Predisposing Characteristics				
Age				
12-13 Years Old (Reference)				
14-15 Years Old	-0.3	5.4	2.2	0.2
	(-6.0,5.4)	(-0.4,11.1)	(-2.2,6.7)	(-3.4,3.8)
16-17 Years Old	-4.6	6.2*	-3.2	-2.7
	(-10.1,0.9)	(0.8,11.6)	(-6.8,0.5)	(-6.4,1.0)
Sex				
Male (Reference)				
Female	12.8***	6.8*	2.6	3.2**
	(8.1,17.4)	(1.2,12.4)	(-0.8,6.0)	(0.8,5.7)
Number of Parents in Household				
Mother or father in household (Reference)				
Mother and father in household	-3.5	-2.0	-1.5	-3.2*
	(-8.0,1.0)	(-6.3,2.3)	(-5.2,2.2)	(-6.3,-0.1)
Other or unknown	-7.3	-0.1	-3.7	-4.9*
	(-16.7,2.1)	(-10.4,10.2)	(-10.1,2.8)	(-9.7,-0.1)

© 2025 Ma Y et al. *JAMA Network Open*.

	Any Mental Health Visit	Any Psychotropic Medication	Any Mental Health Support Group	Any Peer Support Specialist / Recovery Coach
	Estimate (95% CI)	Estimate (95% CI)	Estimate (95% CI)	Estimate (95% CI)
Enabling Characteristics				
Family Income				
<$20,000 (Reference)				
$20,000 - $49,999	0.9 (-6.2,8.0)	-0.6 (-7.9,6.7)	-1.2 (-7.7,5.3)	3.5* (0.1,6.9)
$50,000 - $74,999	3.2 (-5.2,11.6)	5.0 (-3.6,13.6)	-6.2 (-13.0,0.5)	2.5 (-1.9,7.0)
$75,000 or more	2.3 (-7.3,11.8)	2.5 (-5.9,10.8)	-6.7 (-13.9,0.5)	0.8 (-3.5,5.1)
Insurance Status				
Any private insurance (Reference)				
Medicaid (no private insurance)	-4.2 (-10.1,1.7)	-1.0 (-6.8,4.8)	-1.8 (-5.6,2.1)	0.2 (-2.8,3.2)
Other insurance (no private, no Medicaid)	-4.5 (-14.6,5.6)	-11.1* (-20.1,-2.1)	-0.4 (-9.2,8.4)	1.0 (-6.4,8.5)
Uninsured	-8.7 (-23.4,6.1)	-20.6*** (-29.0,-12.3)	-3.1 (-10.1,3.9)	-3.1 (-8.3,2.2)
English Proficiency				
Not very well (Reference)				
Very well	-27.2 (-75.6,21.2)	-14.6 (-59.7,30.5)	7.3 (-39.8,54.5)	-6.7*** (-9.6,-3.9)
Unknown/Missing	0.7 (-6.5,8.0)	-3.4 (-11.4,4.6)	0.4 (-4.5,5.3)	1.6 (-1.3,4.5)
Metropolitan Area				
Large metropolitan area (Reference)				
Small metropolitan area	-1.5 (-5.9,3.0)	1.7 (-2.6,6.0)	-2.0 (-4.8,0.9)	1.3 (-1.1,3.7)
Non-metropolitan area	-5.6 (-11.6,0.3)	-1.7 (-5.5,2.0)	-4.8* (-8.6,-1.0)	0.6 (-3.0,4.2)

© 2025 Ma Y et al. *JAMA Network Open*.

	Any Mental Health Visit	Any Psychotropic Medication	Any Mental Health Support Group	Any Peer Support Specialist / Recovery Coach
	Estimate (95% CI)	Estimate (95% CI)	Estimate (95% CI)	Estimate (95% CI)
Need-related Characteristics				
Self-rated Health				
Good, very good, or excellent (Reference)				
Fair or poor	11.1** (4.4,17.8)	13.3*** (6.6,20.1)	9.8** (3.1,16.5)	4.8* (0.3,9.3)
Any Self-reported Chronic Conditions				
No (Reference)				
Yes	11.0*** (6.1,16.0)	8.6** (2.8,14.4)	1.9 (-1.9,5.8)	1.1 (-1.2,3.4)
Past Year Substance Use Disorder				
No (Reference)				
Yes	11.3*** (6.3,16.3)	17.4*** (12.0,22.8)	4.6* (0.7,8.5)	5.4** (2.0,8.8)
Year	-1.3 (-4.8,2.3)	0.2 (-4.8,5.2)	-1.3 (-3.7,1.2)	1.2 (-1.1,3.5)

Notes: * p<0.05, ** p<0.01, *** p<0.001.
Adjusted probabilities and marginal effects were presented in percentage points. Adjusted probabilities for non-Hispanic Whites were estimated using weighted logistic regression models and evaluated at the observed values of the covariates with race/ethnicity set as non-Hispanic Whites. Marginal effects for each racial/ethnic minority group relative to non-Hispanic Whites were estimated using the same model and evaluated at the observed values of the covariates with non-Hispanic White as the reference group. All estimates were presented with 95% confidence intervals and assessed using two-tailed tests.

© 2025 Ma Y et al. *JAMA Network Open*.

eTable 5 Racial/ethnic differences in past-year mental health service use among all adolescents, by treatment setting

	Outpatient (clinical)	School	Telehealth	Inpatient / residential	Emergency department
	Estimate (95% CI)	Estimate (95% CI)	Estimate (95% CI)	Estimate (95% CI)	Estimate (95% CI)
**Adjusted Probability for White**	21.0***	14.3***	17.0***	3.2***	2.8***
	(19.8,22.1)	(13.2,15.4)	(16.0,18.0)	(2.7,3.7)	(2.3,3.3)
**Marginal Effects**					
Race/Ethnicity					
Asian, Hawaiian, or other Pacific Islander	-8.7***	-2.2	-8.9***	-1.3	-0.7
	(-11.1,-6.3)	(-5.6,1.1)	(-11.8,-6.0)	(-2.9,0.2)	(-2.4,1.0)
Black	-8.1***	-4.0***	-8.4***	0.6	0.4
	(-10.6,-5.7)	(-5.9,-2.0)	(-10.5,-6.4)	(-0.4,1.6)	(-1.0,1.7)
Hispanic	-3.7***	-2.8**	-5.0***	-0.2	-0.6
	(-5.8,-1.7)	(-4.5,-1.0)	(-7.0,-3.0)	(-1.2,0.8)	(-1.6,0.5)
Other	-3.5*	1.0	-1.5	1.0	1.2
	(-6.8,-0.2)	(-2.1,4.2)	(-5.0,1.9)	(-0.8,2.9)	(-0.7,3.0)
Predisposing Characteristics					
Age					
12-13 Years Old (Reference)					
14-15 Years Old	-0.3	-3.5**	2.0	-1.0	-0.6
	(-2.5,2.0)	(-6.1,-1.0)	(-0.1,4.0)	(-2.0,0.0)	(-1.7,0.5)
16-17 Years Old	-2.2	-7.9***	2.2*	-2.4***	-1.7***
	(-4.7,0.2)	(-10.0,-5.8)	(0.0,4.3)	(-3.3,-1.5)	(-2.5,-0.8)
Sex					
Male (Reference)					
Female	7.3***	5.0***	5.5***	0.4	0.8**
	(5.9,8.6)	(3.2,6.8)	(3.7,7.2)	(-0.3,1.0)	(0.2,1.3)
Number of Parents in Household					
Mother or father in household (Reference)					
Mother and father in household	-2.8*	-1.8	-3.5**	-0.4	-0.3
	(-5.1,-0.5)	(-3.8,0.2)	(-5.9,-1.2)	(-1.3,0.5)	(-1.3,0.6)
Other or unknown	-2.6	-3.1	-2.3	-0.4	-0.3
	(-6.6,1.4)	(-7.0,0.9)	(-6.2,1.6)	(-1.6,0.9)	(-1.5,0.9)

© 2025 Ma Y et al. *JAMA Network Open*.

	Outpatient (clinical)	School	Telehealth	Inpatient / residential	Emergency department
	Estimate (95% CI)	Estimate (95% CI)	Estimate (95% CI)	Estimate (95% CI)	Estimate (95% CI)
Enabling Characteristics					
Family Income					
<$20,000 (Reference)					
$20,000 - $49,999	0.9	0.2	0.7	0.4	0.4
	(-1.8,3.6)	(-2.3,2.7)	(-2.4,3.9)	(-0.6,1.4)	(-0.6,1.4)
$50,000 - $74,999	4.3*	1.5	3.5*	-1.0	-0.3
	(0.7,7.8)	(-1.6,4.6)	(0.8,6.2)	(-2.0,0.0)	(-1.4,0.8)
$75,000 or more	3.3*	0.3	4.3**	-0.4	-0.8
	(0.1,6.5)	(-2.6,3.2)	(1.3,7.2)	(-1.5,0.7)	(-2.1,0.4)
Insurance Status					
Any private insurance (Reference)					
Medicaid (no private insurance)	-2.4*	-0.1	-2.3	0.5	-0.2
	(-4.6,-0.1)	(-2.2,1.9)	(-4.6,0.1)	(-0.5,1.5)	(-1.1,0.6)
Other insurance (no private, no Medicaid)	-4.0*	-2.0	-2.1	-0.3	-0.3
	(-7.8,-0.1)	(-5.2,1.2)	(-6.5,2.2)	(-2.0,1.5)	(-2.0,1.4)
Uninsured	-8.3***	-4.4*	-7.5***	-0.1	-2.2***
	(-11.5,-5.1)	(-8.3,-0.4)	(-11.0,-4.0)	(-1.9,1.8)	(-3.2,-1.3)
English Proficiency					
Not very well (Reference)					
Very well	1.9	-0.4	0.7	-1.1	-1.6*
	(-0.8,4.6)	(-3.1,2.3)	(-1.9,3.3)	(-2.3,0.1)	(-3.0,-0.1)
Unknown/Missing	5.9	-2.2	8.5*	3.2	0.8
	(-4.2,16.1)	(-10.6,6.1)	(0.4,16.6)	(-1.2,7.6)	(-3.1,4.7)
Metropolitan Area					
Large metropolitan area (Reference)					
Small metropolitan area	-0.1	-2.3**	-2.4**	0.1	0.2
	(-1.7,1.5)	(-3.9,-0.8)	(-4.2,-0.7)	(-0.6,0.9)	(-0.5,1.0)
Non-metropolitan area	-0.4	-3.9***	-4.9***	0.3	-0.3
	(-3.5,2.7)	(-5.6,-2.2)	(-7.4,-2.4)	(-0.6,1.2)	(-1.3,0.7)

© 2025 Ma Y et al. *JAMA Network Open*.

	Outpatient (clinical)	School	Telehealth	Inpatient / residential	Emergency department
	Estimate (95% CI)	Estimate (95% CI)	Estimate (95% CI)	Estimate (95% CI)	Estimate (95% CI)
Need-related Characteristics					
Major Depressive Episode in life time					
No (Reference)					
Yes	20.1***	17.0***	19.8***	3.1***	4.4***
	(18.0,22.1)	(14.9,19.1)	(17.3,22.4)	(2.2,3.9)	(3.3,5.5)
Unknown/Missing	5.5*	1.7	6.4*	0.5	1.5
	(0.4,10.6)	(-2.0,5.3)	(1.3,11.5)	(-0.7,1.8)	(-0.5,3.5)
Self-rated Health					
Good, very good, or excellent (Reference)					
Fair or poor	8.8***	5.8***	6.6***	2.7**	1.5*
	(5.0,12.5)	(3.2,8.5)	(3.0,10.1)	(0.9,4.6)	(0.3,2.8)
Any Self-reported Chronic Conditions					
No (Reference)					
Yes	6.7***	3.6***	5.3***	1.4**	0.7
	(4.7,8.8)	(1.6,5.6)	(3.4,7.2)	(0.5,2.3)	(-0.1,1.6)
Past Year Substance Use Disorder					
No (Reference)					
Yes	6.8***	6.1***	9.4***	5.8***	4.5***
	(4.1,9.6)	(4.0,8.2)	(6.4,12.4)	(3.8,7.7)	(2.7,6.2)
Year	0.0	0.6	0.4	0.2	-0.2
	(-1.8,1.7)	(-0.8,2.0)	(-1.2,2.1)	(-0.5,1.0)	(-0.9,0.5)

Notes: * $p<0.05$, ** $p<0.01$, *** $p<0.001$.
Adjusted probabilities and marginal effects were presented in percentage points. Adjusted probabilities for non-Hispanic Whites were estimated using weighted logistic regression models and evaluated at the observed values of the covariates with race/ethnicity set as non-Hispanic Whites. Marginal effects for each racial/ethnic minority group relative to non-Hispanic Whites were estimated using the same model and evaluated at the observed values of the covariates with non-Hispanic White as the reference group. All estimates were presented with 95% confidence intervals and assessed using two-tailed tests.

© 2025 Ma Y et al. *JAMA Network Open*.

eTable 6 Racial/ethnic differences in past-year mental health service use among adolescents with past major depressive episodes, by treatment setting

	Outpatient (clinical)	School	Telehealth	Inpatient / residential	Emergency department
	Estimate (95% CI)	Estimate (95% CI)	Estimate (95% CI)	Estimate (95% CI)	Estimate (95% CI)
**Adjusted Probability for NH White**	41.1***	29.3***	36.8***	6.6***	7.0***
	(38.2,44.0)	(26.8,31.9)	(33.8,39.8)	(5.4,7.8)	(5.6,8.4)
**Marginal Effects**					
Race/Ethnicity					
Asian, Hawaiian, or other Pacific Islander	-19.8***	-3.2	-16.2***	0.2	-1.2
	(-28.9,-10.8)	(-14.1,7.7)	(-24.4,-8.0)	(-6.3,6.7)	(-7.6,5.3)
Black	-18.5***	-11.6***	-19.0***	-0.6	-0.3
	(-24.9,-12.1)	(-16.2,-7.0)	(-24.6,-13.4)	(-3.7,2.5)	(-4.1,3.5)
Hispanic	-7.8**	-3.5	-11.1***	-1.2	-1.1
	(-12.7,-2.9)	(-8.6,1.5)	(-16.1,-6.1)	(-3.7,1.3)	(-3.8,1.7)
Other	-7.5	-1.6	-4.7	0.6	2.6
	(-16.2,1.2)	(-8.4,5.2)	(-14.1,4.8)	(-4.6,5.8)	(-3.2,8.5)
Predisposing Characteristics					
Age					
12-13 Years Old (Reference)					
14-15 Years Old	6.3*	-5.9	8.7**	-0.1	-3.0
	(1.2,11.3)	(-12.4,0.6)	(2.8,14.6)	(-3.6,3.5)	(-6.2,0.2)
16-17 Years Old	3.5	-14.5***	5.1*	-4.3**	-5.6***
	(-1.7,8.7)	(-20.4,-8.5)	(0.0,10.2)	(-7.0,-1.5)	(-8.4,-2.9)
Sex					
Male (Reference)					
Female	16.7***	7.2**	8.0**	1.8	2.1*
	(13.0,20.5)	(2.4,11.9)	(2.7,13.2)	(-0.3,3.8)	(0.1,4.1)
Household Structure					
Mother or father in household (Reference)					
Mother and father in household	-0.1	-1.1	-2.8	-0.5	-2.3
	(-5.6,5.4)	(-5.8,3.5)	(-7.4,1.8)	(-3.4,2.4)	(-5.3,0.8)
Other or unknown	-4.1	-13.3**	-2.6	0.1	0.9
	(-13.8,5.6)	(-21.3,-5.4)	(-12.2,7.1)	(-3.9,4.2)	(-3.7,5.4)

© 2025 Ma Y et al. *JAMA Network Open*.

	Outpatient (clinical)	School	Telehealth	Inpatient / residential	Emergency department
	Estimate (95% CI)	Estimate (95% CI)	Estimate (95% CI)	Estimate (95% CI)	Estimate (95% CI)
Enabling Characteristics					
Family Income					
<$20,000 (Reference)					
$20,000 - $49,999	-0.2 (-7.4,7.1)	-4.4 (-12.0,3.2)	-1.6 (-9.2,6.1)	4.0* (0.9,7.1)	3.7* (0.7,6.8)
$50,000 - $74,999	3.5 (-4.8,11.9)	-2.3 (-10.9,6.3)	5.9 (-2.5,14.3)	-0.7 (-3.5,2.1)	2.0 (-1.0,5.0)
$75,000 or more	3.2 (-6.0,12.5)	-1.4 (-9.8,6.9)	4.0 (-4.8,12.8)	0.7 (-2.4,3.9)	1.3 (-2.1,4.8)
Insurance Status					
Any private insurance (Reference)					
Medicaid (no private insurance)	-3.6 (-9.4,2.3)	-0.2 (-5.6,5.2)	-3.5 (-9.0,2.0)	0.2 (-2.4,2.7)	0.0 (-2.8,2.8)
Other insurance (no private, no Medicaid)	-3.4 (-14.5,7.6)	-2.7 (-9.0,3.6)	-2.2 (-14.1,9.6)	-0.6 (-5.9,4.7)	-0.4 (-5.9,5.1)
Uninsured	-11.2 (-24.2,1.8)	-0.8 (-15.8,14.1)	-15.5* (-28.6,-2.5)	-2.7 (-8.1,2.6)	-6.0*** (-8.6,-3.4)
English Proficiency					
Not very well (Reference)					
Very well	3.8 (-3.2,10.9)	0.1 (-6.8,7.0)	-1.7 (-8.9,5.5)	-2.0 (-5.2,1.2)	-2.2 (-5.5,1.2)
Unknown/Missing	-6.2 (-56.4,43.9)	-29.3*** (-36.0,-22.5)	-10.9 (-59.4,37.5)	16.6 (-29.8,62.9)	16.7 (-33.0,66.5)
Metropolitan Area					
Large metropolitan area (Reference)					
Small metropolitan area	-0.2 (-4.3,3.8)	-5.2** (-8.8,-1.5)	-4.2* (-7.9,-0.5)	1.0 (-1.1,3.2)	0.3 (-2.1,2.7)
Non-metropolitan area	-1.8 (-6.4,2.8)	-6.7* (-12.0,-1.3)	-9.2*** (-14.6,-3.8)	0.7 (-2.1,3.5)	-2.2 (-4.6,0.2)

© 2025 Ma Y et al. *JAMA Network Open*.

	Outpatient (clinical)	School	Telehealth	Inpatient / residential	Emergency department
	Estimate (95% CI)	Estimate (95% CI)	Estimate (95% CI)	Estimate (95% CI)	Estimate (95% CI)
Need-related Characteristics					
Self-rated Health					
Good, very good, or excellent (Reference)					
Fair or poor	12.8***	10.7***	8.2*	5.7**	2.9
	(5.6,19.9)	(4.5,16.9)	(1.2,15.2)	(1.7,9.7)	(0.0,5.9)
Any Self-reported Chronic Conditions					
No (Reference)					
Yes	9.3***	3.4	7.6**	1.9	1.3
	(4.5,14.1)	(-1.4,8.2)	(2.8,12.4)	(-0.3,4.0)	(-1.1,3.7)
Past Year Substance Use Disorder					
No (Reference)					
Yes	9.5***	10.7***	14.0***	9.9***	9.7***
	(4.1,14.9)	(5.8,15.5)	(8.5,19.4)	(5.7,14.1)	(6.0,13.3)
Year	-1.6	-0.7	-1.8	-0.4	-1.5
	(-6.5,3.3)	(-4.7,3.2)	(-6.0,2.4)	(-2.7,1.9)	(-3.5,0.6)

Notes: * $p<0.05$, ** $p<0.01$, *** $p<0.001$.
Adjusted probabilities and marginal effects were presented in percentage points. Adjusted probabilities for non-Hispanic Whites were estimated using weighted logistic regression models and evaluated at the observed values of the covariates with race/ethnicity set as non-Hispanic Whites. Marginal effects for each racial/ethnic minority group relative to non-Hispanic Whites were estimated using the same model and evaluated at the observed values of the covariates with non-Hispanic White as the reference group. All estimates were presented with 95% confidence intervals and assessed using two-tailed tests.

© 2025 Ma Y et al. *JAMA Network Open*.

eFigure 1 Adjusted racial/ethnic differences in past-year mental health service use among adolescents with past major depressive disorder, by treatment type, 2022-2023

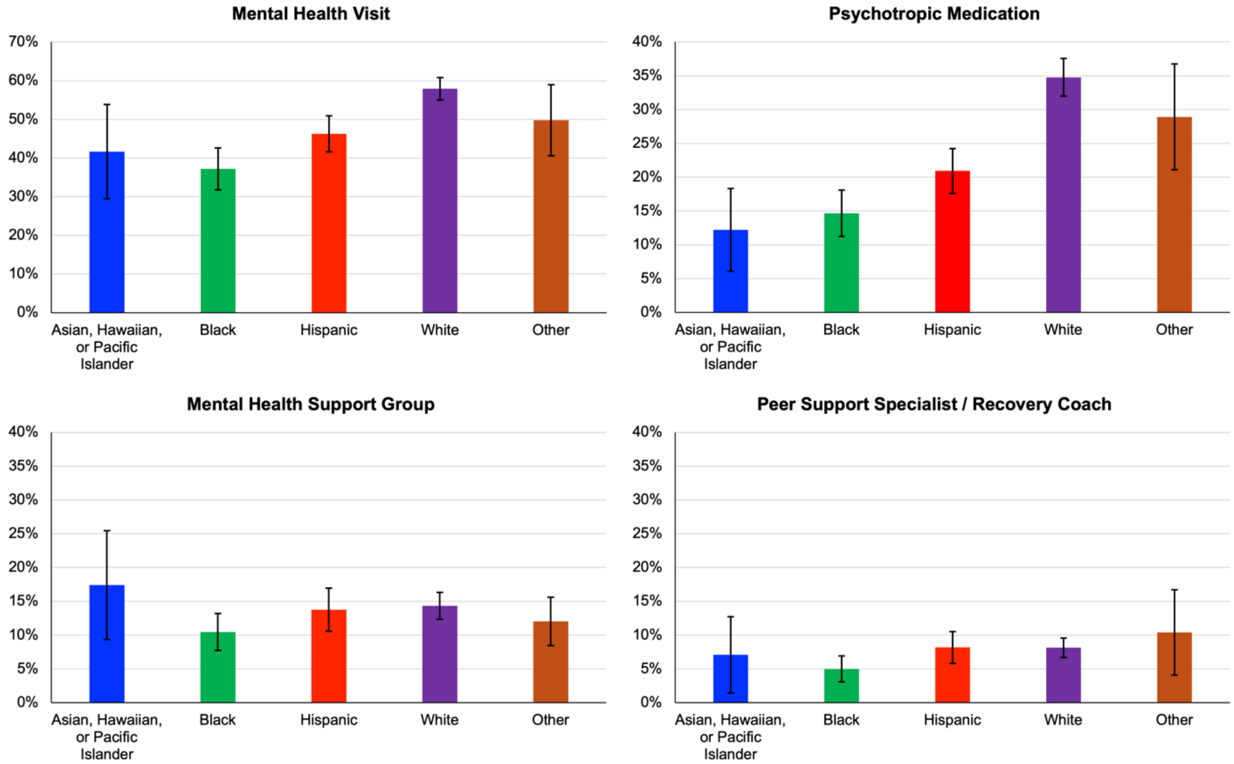

Notes:
Analysis conducted among adolescents with past major depressive episode. Adjusted probabilities for each race/ethnicity were estimated using weighted logistic regression models and evaluated at the observed values of the covariates with race/ethnicity set as the corresponding race/ethnicity category. Separate logistic regression models were estimated for each treatment type. All estimates were presented with 95% confidence intervals.

© 2025 Ma Y et al. *JAMA Network Open*.

eFigure 2 Adjusted racial/ethnic differences in past-year mental health service use among adolescents with past major depressive disorder, by treatment setting, 2022-2023

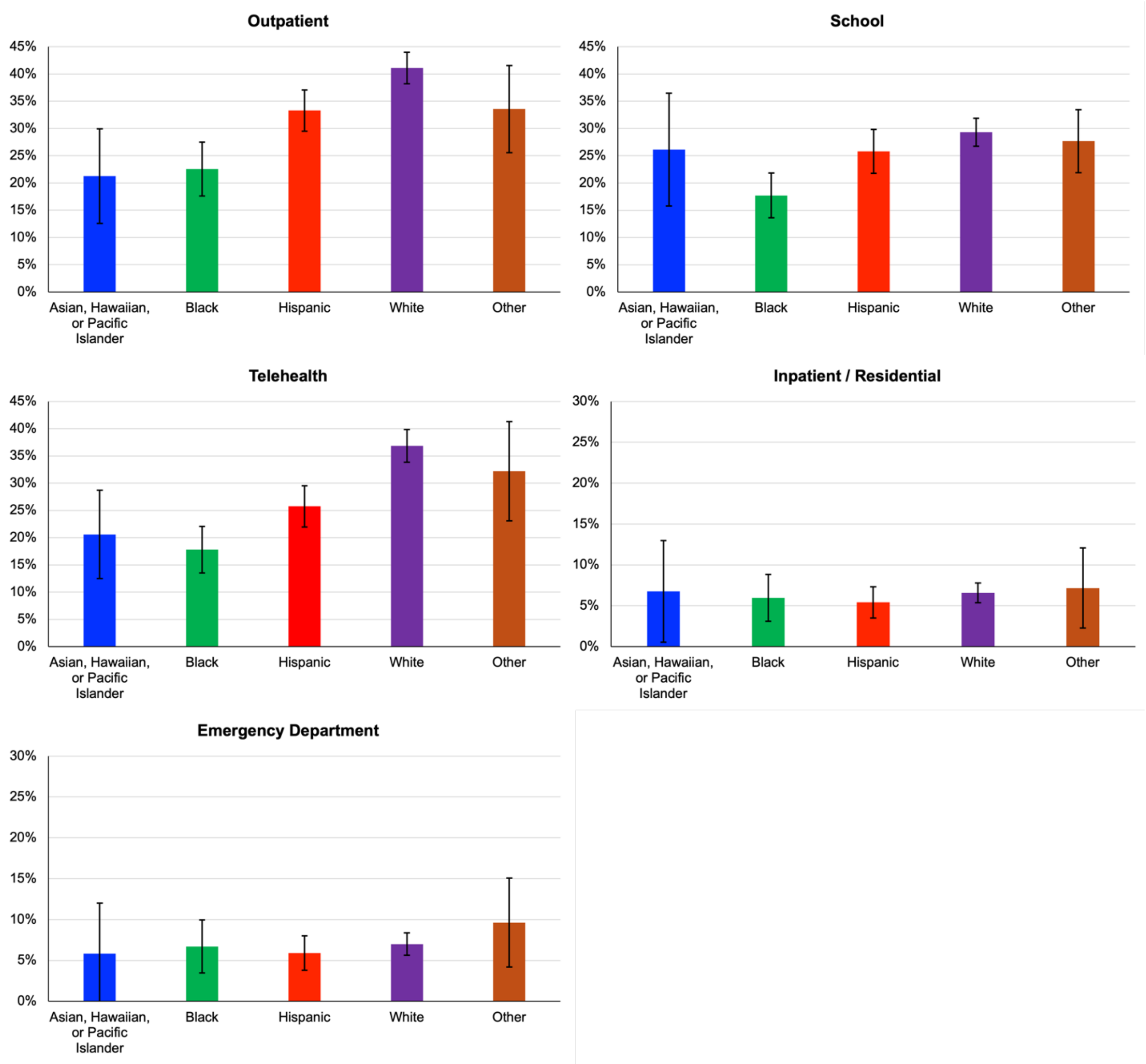

Notes:
Analysis conducted among adolescents with past major depressive episode. Adjusted probabilities for each race/ethnicity were estimated using weighted logistic regression models and evaluated at the observed values of the covariates with race/ethnicity set as the corresponding race/ethnicity category. Separate logistic regression models were estimated for each treatment setting. All estimates were presented with 95% confidence intervals.

© 2025 Ma Y et al. *JAMA Network Open*.
