# Supplementary material for: Racial and Ethnic Differences in Mental Health Service Use Among Adolescents
Source: JAMA Netw Open. 2025 Jun 18;8(6):e2516612. doi: 10.1001/jamanetworkopen.2025.16612 (PMC12177673; doi:10.1001/jamanetworkopen.2025.16612)
Supplement: Supplement 2. — Data Sharing Statement [file jamanetwopen-e2516612-s002.pdf]

Data Sharing Statement

Ma. Racial and Ethnic Differences in Mental Health Service Use Among Adolescents. *JAMA Netw Open*. Published online June 18, 2025. doi:10.1001/jamanetworkopen.2025.16612

Data
Data available: No
